# Crystal structure of hexa­sodium tetra­serinolium paratungstate B deca­hydrate, [Na_6_{(CH_2_OH)_2_CHNH_3_}_4_][W_12_O_40_(OH)_2_]·10H_2_O

**DOI:** 10.1107/S2056989022000457

**Published:** 2022-01-18

**Authors:** Kleanthi Sifaki, Nadiia I. Gumerova, Gerald Giester, Annette Rompel

**Affiliations:** a Universität Wien, Fakultät für Chemie, Institut für Biophysikalische Chemie, Althanstr. 14, Wien 1090, Austria; b Universität Wien, Fakultät für Geowissenschaften, Geographie und Astronomie, Institut für Mineralogie und Kristallographie, Althanstr. 14, Wien 1090, Austria

**Keywords:** crystal structure, metal oxide, polyoxometalate, isopolytungstate, organic–inorganic hybrid, alkoxo ligand

## Abstract

The title polyoxometalate-based organic–inorganic hybrid compound, [Na_6_(C_3_H_10_NO_2_)_4_][W_12_O_40_(OH)_2_]·10H_2_O, consists of a di-*μ*_3_-hydroxido-tetra-*μ*_3_-oxido-octa­deca-*μ*-oxido-octa­deca­oxidododeca­tungstate (para­do­deca­tungstate B) anion, [W_12_O_40_(OH)_2_]^10–^, and six sodium cations coordinated by the oxygen ions of the polyanions, serinol ligands protonated at the N atom, and water mol­ecules. The centrosymmetric paratungstate B anion shows characteristic features in terms of bond lengths and angles. The three-dimensional framework structure is established by bonding of the sodium cations with oxygen ions of the paratungstate B anions and a network consisting of N—H⋯O and O—H⋯O hydrogen bonds of medium strength between the protonated serinol cations, water mol­ecules and the paratungstate B anions. The title compound was also characterized by means of elemental analysis, IR spectroscopy and thermogravimetric analysis.

## Abstract 2

The isolation and characterization of an isopolytungstate anion [W_12_O_40_(OH)_2_]^10–^ (paradodeca­tungstate B) with sodium and protonated serinol entities [Na_6_((CH_2_OH)_2_CHNH_3_)_4_]^10+^ as counter-cations are reported.

## Chemical context

Polyoxometalates (POMs) are discrete anionic mol­ecular clusters of metal oxide entities, which usually consist of transition metals of groups V and VI in their highest oxidation states. POMs exist at a unique inter­face between monomeric oxometalates and polymeric metal oxides and have a wide range of applications (Pope, 1983 ▸; Gumerova & Rompel, 2020 ▸). To date, a variety of strategies have been developed and used to build POM-based hybrid materials by varying the reaction conditions such as the type of addenda ions, organic ligands, pH, solvents, the molar ratio of the starting materials or the reaction environments. The [W_12_O_40_(OH)_2_]^10–^ paratungstate B anion is stable in aqueous acidic solution and has a cluster-like structure of twelve W-centered distorted octa­hedra {WO_6_} (Evans & Rollins, 1976 ▸; Pope, 1983 ▸). Due to its high surface charge density *q/m* = 0.83 (*q* = net charge, *m* = number of metal ions), the paratungstate B anion can act as a multidentate ligand for alkaline (Peresypkina *et al.*, 2014 ▸) or transition-metal cations (Radio *et al.*, 2010 ▸, 2011 ▸; Gumerova *et al.*, 2015 ▸, 2018 ▸) and as a precursor for the synthesis of catalytically active sandwich-type polyoxotungstates (POTs) (Sokolov *et al.*, 2012 ▸).

A search in the Cambridge Structural Database (version 5.42, update of November 2021; Groom *et al.*, 2016 ▸) revealed that seventeen organic–inorganic hybrid paratungstates B have been structurally characterized so far. We are expanding the class of hybrid paratungstates by using serinol (C_3_H_9_NO_2_; 2-amino-1,3-propandiol), which has not previously been used in its protonated form as a counter-cation for paratungstates and can coordinate to metal cations in different ways *via* its –NH_2_ or HOCH_2_– groups and thus influences both the structure and the properties of the compound significantly (Sifaki *et al.*, 2021 ▸). Serinol is a very stable, readily water-soluble, non-toxic, odorless, biodegradable compound that is used as a versatile starting material in organic synthesis and as an additive for material applications, such as composite materials (Barbera *et al.*, 2020 ▸; Andreessen & Steinbüchel, 2011 ▸). In POM synthesis, due to its amino group, serinol can be regarded as an alk­oxy­lation ligand and/or as a buffer compound (p*K_a_* = 12.2; Chemicalbook, 2021 ▸). With its protonated amine group it can also act as a counter-cation. 
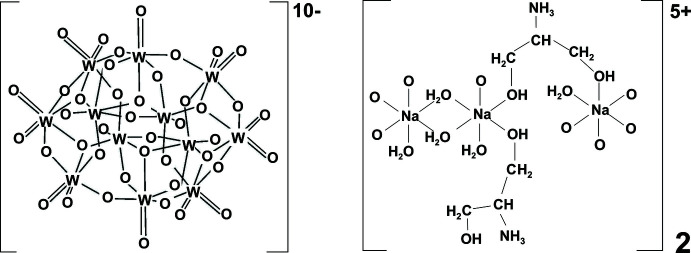


## Structural commentary

**Figure 1:**
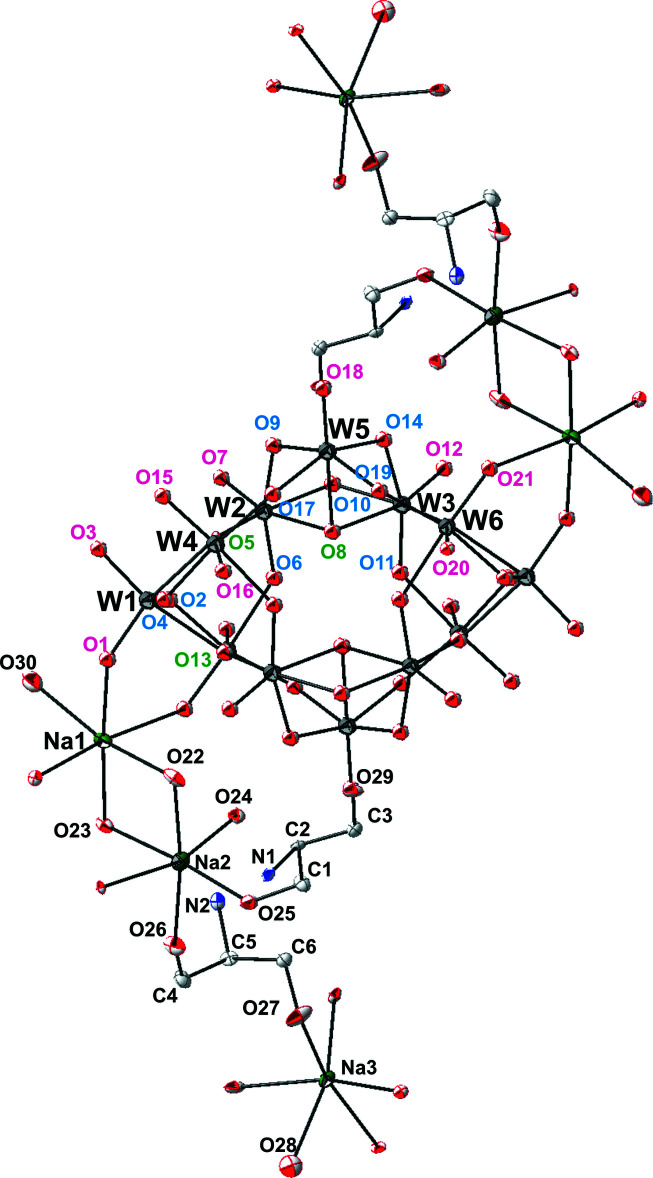
The principal building units in the crystal structure of [Na_6_((CH_2_OH)_2_CHNH_3_)_4_][W_12_O_40_(OH)_2_]·10H_2_O. The asymmetric unit was doubled considering the inversion center, and coordination spheres for all cations were completed. Labeling of all atoms of asymmetric unit is shown; non-labeled atoms are generated by symmetry operation −*x* + 1, −*y* + 1, −*z* + 1. The oxygen atoms in the paratungstate B anion are labeled according to their coordination mode: magenta for the terminal, blue for the double bridging, green for the triply bridging oxygen ions. Displacement ellipsoids are drawn at the 50% probability level. Color code: W, dark gray; Na, green; C, gray; N, blue; O, red.

**Figure 2:**
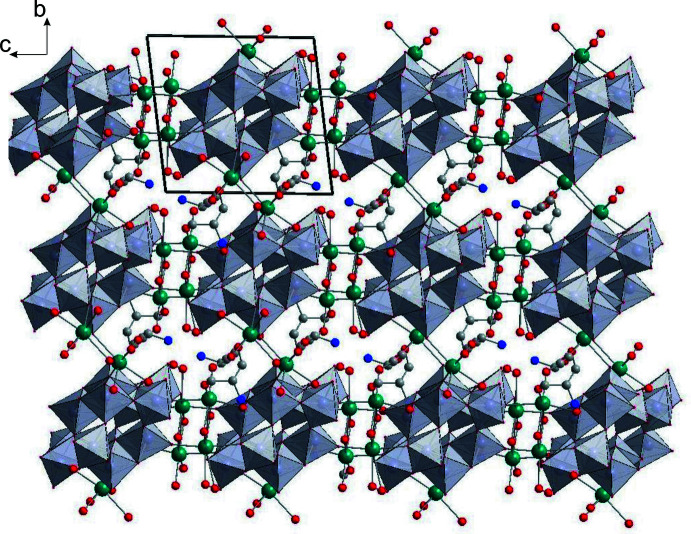
The crystal packing of [Na_6_((CH_2_OH)_2_CHNH_3_)_4_][W_12_O_40_(OH)_2_]·10H_2_O viewed along the *a* axis. Color code: {WO_6_}: gray octa­hedra; Na: green; C: gray; N: blue; O: red.

The asymmetric unit of the title compound is made up of half of the paratungstate B polyoxoanion, [W_12_O_40_(OH)_2_]^10–^, three sodium cations coordinated by water mol­ecules, terminal oxygen atoms of the paratungstate anion and the oxygen atoms of HOCH_2_– groups from two serinol cations protonated at the N atoms [(CH_2_OH)_2_CHNH_3_^+^] (Fig. 1 ▸). An inversion center creates a full unit with formula [Na_6_((CH_2_OH)_2_CHNH_3_)_4_][W_12_O_40_(OH)_2_]·10H_2_O (Fig. 2 ▸). The centrosymmetric paratungstate B anion [W_12_O_40_(OH)_2_]^10–^ is structurally very similar to previously described ones (Radio *et al.*, 2010 ▸, 2011 ▸; Gumerova *et al.*, 2015 ▸, 2018 ▸) and consists of four groups, *viz*. two {W_3_O_13_} and two {W_3_O_14_} units, with common vertices (Fig. 1 ▸). In the {W_3_O_13_} groups each {WO_6_} octa­hedron has a terminal oxygen atom, while in the {W_3_O_14_} units each {WO_6_} octa­hedron has two unshared oxygen ions (Fig. 1 ▸). The oxygen atoms associated with the central W ions can be divided into three groups: 1) terminal oxygen ions (O_t_), each bonded to a W ion (magenta labeling in Fig. 1 ▸); 2) bridging oxygen ions (O_db_), each connected to two W ions (blue labeling in Fig. 1 ▸); 3) triply bridging oxygen ions (O_tb_) linked to three W ions (green labeling in Fig. 1 ▸).

## Supra­molecular features

The paratungstate B anion is bound to twelve Na^+^ cations *via* both terminal (O_t_) and bridging oxygen atoms (O_db_). Each of the surrounding four Na1 cations and six Na3 cations are coordinated by the O atoms of two polyanions, while two Na2 cations are bound to one terminal oxygen atom of the polyanion and to the O atoms of two serinolium cations, which are further connected to the Na3 cations *via* the HOCH_2_– groups. Thus, a three-dimensional framework is established in the crystal structure by connecting paratungstate B anions through Ot—Na1—Ot, Ot—Na3—Ot and Ot—Na2–serinolium—Na3—Ot bridges (Fig. 2 ▸). An intimate network of N—H⋯O and O—H⋯O hydrogen bonds of medium strength between the protonated serinol ligands, polyoxoanions and water mol­ecules consolidates the crystal packing (Table 1 ▸).

## Database survey

A search in the Cambridge Structural Database (CSD; version 5.42, update of November 2021; Groom *et al.*, 2016 ▸) indicated 17 structures with the formula search [‘W_12_O_42_’ or ‘W_12_O_40_(OH)_2_’], including eleven structures with only organic cations and six structures with organic and transition-metal cations. The 17 compounds deposited in the CSD all crystallize in centrosymmetric space groups, and the bond lengths in the paratungstate B anion are very similar to those observed in the title structure. A similar structure containing sodium and a protonated imidazole as counter-cations, *viz*. Na_2_(HIm)_8_[W_12_O_40_(OH)_2_]·10H_2_O (HIm: imidazolium), is comprised of infinite inorganic chains built up from [W_12_O_40_(OH)_2_]^10–^ anions and sodium cations. Adjacent chains are further connected by hydrogen-bonding inter­actions between imid­azolium cations, water mol­ecules, and polyoxoanions (Chaalia *et al.*, 2012 ▸).

## Synthesis and crystallization

To obtain the title compound, Na_2_WO_4_·2H_2_O (0.495 g, 1.5 mmol) was dissolved in 5 ml of distilled water and acidified with 1 *M* HCl to pH 3. Serinol (0.075 g, 0.8 mmol) was then added to the acidified orthotungstate solution, which increased the pH to 6.7. The reaction mixture was then heated to 363 K and stirred for 1 h, cooled to room temperature and left covered with parafilm. Colorless block-shaped crystals were filtered off after one week from the mother liquor, washed with water and ethanol and then air-dried (yield 0.12 g; 27%, based on W). Elemental analysis (%) for C_12_H_62_N_4_Na_6_O_60_W_12_ (calculated): C 3.23 (4.04), H 1.71 (1.75), N 1.41 (1.57), O 26.68 (26.92). FT–IR (cm^−1^): 3340 (*s*), 2952 (*sh*), 2889 (*sh*), 1614 (*s*), 1450 (*m*), 1066 (*w*), 1037 (*m*), 996 (*w*), 930 (*s*), 896 (*s*), 794 (*s*), 681 (*s*), 620 (*m*), 478 (*s*), 456 (*s*), 428 (*s*), 310 (*s*). Mass loss observed in thermogravimetric analysis in the temperature range 298–1073 K (calculated for four protonated serinol ligands, ten crystal water mol­ecules and one water mol­ecule from the anion): 16.67% (16.78%).

## Refinement

Crystal data, data collection and structure refinement details are summarized in Table 2 ▸. The positions of the H atoms of the water mol­ecules (O22, O23, O24, O28) were obtained by difference-Fourier techniques and were refined with free isotropic displacement parameters and O—H distances restrained to 0.95 (2) Å. The disordered water mol­ecule (O30) was refined with two positions (O30*A* and O30*B*), with free occupancy factors to a total of 100%. H atoms bound to N or C atoms were placed in idealized positions (N—H = 0.91 Å and C—H = 0.99 or 1.00 Å for CH_2_ and CH groups, respectively) and refined in riding modes, with *U*_iso_(H) values set to 1.5*U*_eq_(N) or to 1.2*U*_eq_(C). Three H-atom positions could not be included in the final model: two H-atom positions from the disordered water mol­ecule (O30*A* and O30*B*), and one H atom that should be located inside the paratungstate B anion on the triply bridging O8 atom, which was previously proven by neutron diffraction (Evans & Prince, 1983 ▸).

## Supplementary Material

Crystal structure: contains datablock(s) I. DOI: 10.1107/S2056989022000457/wm5630sup1.cif

Structure factors: contains datablock(s) I. DOI: 10.1107/S2056989022000457/wm5630Isup2.hkl

CCDC reference: 2121767

Additional supporting information:  crystallographic information; 3D view; checkCIF report

## Figures and Tables

**Table 1:** Hydrogen-bond geometry (Å, °)

*D*—H⋯*A*	*D*—H	H⋯*A*	*D*⋯*A*	*D*—H⋯*A*
O22—H22*A*⋯O1^i^	0.67 (7)	2.11 (7)	2.757 (5)	163 (8)
O23—H23*A*⋯O16^i^	0.78 (7)	2.04 (7)	2.802 (5)	165 (7)
O23—H23*B*⋯O16^ii^	0.91 (7)	1.91 (7)	2.811 (5)	175 (6)
O25—H25⋯O3^iii^	0.73 (6)	2.05 (7)	2.761 (5)	164 (7)
O26—H26⋯O15^i^	0.88 (7)	1.88 (7)	2.730 (5)	160 (6)
O27—H27⋯O2^iii^	0.67 (7)	2.04 (7)	2.696 (5)	165 (8)
O28—H28*A*⋯O21^iv^	0.75 (8)	2.01 (8)	2.735 (6)	160 (8)
O29—H29⋯O10^v^	0.81 (6)	1.91 (7)	2.692 (5)	163 (6)
N1—H1*B*⋯O15^iii^	0.91	1.82	2.723 (5)	170
N2—H2*B*⋯O24	0.91	1.96	2.867 (6)	172

Symmetry codes: (i) -x+1, -y+1, -z; (ii) x-1, y, z; (iii) x, y-1, z; (iv) -x+1, -y, -z+1; (v) -x+1, -y+1, -z+1.

**Table 2:** Experimental details

Crystal data	
Chemical formula	[Na_6_((C_12_H_40_N_4_O_8_)][W_12_O_40_(OH)_2_]·10H_2_O
*M*_r_	3566.79
Crystal system, space group	Triclinic, *P*\overline{1}
Temperature (K)	200
*a*, *b*, *c* (Å)	12.0541 (8), 12.0821 (8), 12.7050 (8)
α, β, γ (°)	73.180 (2), 65.308 (2), 64.345 (2)
*V* (Å^3^)	1502.07 (17)
*Z*	1
Radiation type	Mo *K*α
μ (mm^−1^)	23.04
Crystal size (mm)	0.05 × 0.05 × 0.05

Data collection	
Diffractometer	Bruker APEXII CCD
Absorption correction	Multi-scan (*SADABS*; Krause *et al.*, 2015 ▸)
*T*_min_, *T*_max_	0.245, 0.747
No. of measured, independent and observed [*I* > 2σ(*I*)] reflections	39702, 5490, 5404
*R*_int_	0.038
(sin θ/λ)_max_ (Å^−1^)	0.602

Refinement	
*R*[*F* ^2^ > 2σ(*F* ^2^)], *wR*(*F* ^2^), *S*	0.017, 0.043, 1.25
No. of reflections	5490
No. of parameters	472
No. of restraints	6
H-atom treatment	H atoms treated by a mixture of independent and constrained refinement
Δρ_max_, Δρ_min_ (e Å^−3^)	0.99, −1.62

Computer programs: *APEX3* and *SAINT* (Bruker, 2016 ▸), *olex2.solve* (Bourhis *et al.*, 2015 ▸), *SHELXL* (Sheldrick, 2015 ▸) and *OLEX2* (Dolomanov *et al.*, 2009 ▸).

## supplementary crystallographic information

### Crystal data

**Table:**

[Na_6_(C_3_H_10_NO_2_)_4_][W_12_O_40_(OH)_2_]·10H_2_O	*Z* = 1
*M**_r_* = 3566.79	*F*(000) = 1596
Triclinic, *P*1	*D*_x_ = 3.943 Mg m^−^^3^
*a* = 12.0541 (8) Å	Mo *K*α radiation, λ = 0.71073 Å
*b* = 12.0821 (8) Å	Cell parameters from 9742 reflections
*c* = 12.7050 (8) Å	θ = 2.2–33.1°
α = 73.180 (2)°	µ = 23.04 mm^−^^1^
β = 65.308 (2)°	*T* = 200 K
γ = 64.345 (2)°	Block, clear colourless
*V* = 1502.07 (17) Å^3^	0.05 × 0.05 × 0.05 mm

### Data collection

**Table:**

Bruker APEXII CCD diffractometer	5404 reflections with *I* > 2σ(*I*)
Radiation source: sealed x-ray tube	*R*_int_ = 0.038
φ and ω scans	θ_max_ = 25.4°, θ_min_ = 2.2°
Absorption correction: multi-scan (*SADABS*; Krause *et al.*, 2015)	*h* = −14→14
*T*_min_ = 0.245, *T*_max_ = 0.747	*k* = −14→14
39702 measured reflections	*l* = −15→15
5490 independent reflections	

### Refinement

**Table:**

Refinement on *F*^2^	Primary atom site location: iterative
Least-squares matrix: full	Hydrogen site location: mixed
*R*[*F*^2^ > 2σ(*F*^2^)] = 0.017	H atoms treated by a mixture of independent and constrained refinement
*wR*(*F*^2^) = 0.043	*w* = 1/[σ^2^(*F*_o_^2^) + (0.0095*P*)^2^ + 5.2022*P*] where *P* = (*F*_o_^2^ + 2*F*_c_^2^)/3
*S* = 1.25	(Δ/σ)_max_ = 0.002
5490 reflections	Δρ_max_ = 0.99 e Å^−^^3^
472 parameters	Δρ_min_ = −1.61 e Å^−^^3^
6 restraints	

### Special details

**Table:**

Geometry. All esds (except the esd in the dihedral angle between two l.s. planes) are estimated using the full covariance matrix. The cell esds are taken into account individually in the estimation of esds in distances, angles and torsion angles; correlations between esds in cell parameters are only used when they are defined by crystal symmetry. An approximate (isotropic) treatment of cell esds is used for estimating esds involving l.s. planes.
Refinement. olex2_refinement_description 1. Fixed Uiso At 1.2 times of: All C(H) groups, All C(H,H) groups, All O(H) groups At 1.5 times of: All N(H,H,H) groups, All O(H,H) groups 2. Uiso/Uaniso restraints and constraints O30A ~ O30B: within 2A with sigma of 0.04 and sigma for terminal atoms of 0.08 within 2A 3. Others Sof(O30A)=1-FVAR(1) Sof(O30B)=FVAR(1) 4.a Ternary CH refined with riding coordinates: C2(H2), C5(H5) 4.b Secondary CH2 refined with riding coordinates: C1(H1D,H1E), C3(H3A,H3B), C4(H4A,H4B), C6(H6A,H6B) 4.c Idealised Me refined as rotating group: N1(H1A,H1B,H1C), N2(H2A,H2B,H2C)

### Fractional atomic coordinates and isotropic or equivalent isotropic displacement parameters (Å^2^)

**Table:**

	*x*	*y*	*z*	*U*_iso_*/*U*_eq_	Occ. (<1)
W1	0.42560 (2)	0.74680 (2)	0.16437 (2)	0.00609 (5)	
W2	0.38107 (2)	0.76530 (2)	0.46722 (2)	0.00492 (5)	
W3	0.43557 (2)	0.58129 (2)	0.71174 (2)	0.00468 (5)	
W4	0.70356 (2)	0.65943 (2)	0.21152 (2)	0.00538 (5)	
W5	0.66091 (2)	0.68816 (2)	0.50890 (2)	0.00550 (5)	
W6	0.79490 (2)	0.36191 (2)	0.62441 (2)	0.00589 (5)	
Na1	0.2506 (2)	0.6297 (2)	0.05508 (18)	0.0188 (4)	
Na2	0.2644 (2)	0.32264 (19)	0.11025 (19)	0.0195 (5)	
Na3	−0.1267 (2)	−0.09506 (18)	0.40382 (18)	0.0166 (4)	
O1	0.3975 (3)	0.7093 (3)	0.0574 (3)	0.0103 (7)	
O2	0.2557 (3)	0.7800 (3)	0.2802 (3)	0.0091 (7)	
O3	0.4137 (3)	0.9003 (3)	0.1124 (3)	0.0119 (7)	
O4	0.6114 (3)	0.6631 (3)	0.1144 (3)	0.0077 (7)	
O5	0.4911 (3)	0.7439 (3)	0.3094 (3)	0.0068 (6)	
O6	0.2927 (3)	0.6703 (3)	0.4788 (3)	0.0098 (7)	
O7	0.2695 (3)	0.9141 (3)	0.4594 (3)	0.0114 (7)	
O8	0.5351 (3)	0.5906 (3)	0.5163 (3)	0.0061 (6)	
O9	0.5209 (3)	0.8146 (3)	0.4727 (3)	0.0070 (6)	
O10	0.3393 (3)	0.7379 (3)	0.6367 (3)	0.0058 (6)	
O11	0.3480 (3)	0.5000 (3)	0.6978 (3)	0.0096 (7)	
O12	0.3556 (3)	0.6163 (3)	0.8530 (3)	0.0108 (7)	
O13	0.5815 (3)	0.4361 (3)	0.7240 (3)	0.0077 (7)	
O14	0.5651 (3)	0.6687 (3)	0.6669 (3)	0.0081 (7)	
O15	0.7266 (3)	0.7992 (3)	0.1354 (3)	0.0116 (7)	
O16	0.8550 (3)	0.5513 (3)	0.1411 (3)	0.0092 (7)	
O17	0.7336 (3)	0.6599 (3)	0.3434 (3)	0.0088 (7)	
O18	0.7542 (3)	0.7663 (3)	0.5051 (3)	0.0113 (7)	
O19	0.7760 (3)	0.5146 (3)	0.5302 (3)	0.0090 (7)	
O20	0.9485 (3)	0.2716 (3)	0.5427 (3)	0.0104 (7)	
O21	0.8303 (3)	0.3935 (3)	0.7318 (3)	0.0113 (7)	
O22	0.3854 (4)	0.4292 (4)	0.1050 (4)	0.0193 (9)	
H22A	0.447 (7)	0.401 (7)	0.068 (6)	0.029*	
H22B	0.397 (7)	0.418 (6)	0.174 (6)	0.029*	
O23	0.1180 (4)	0.5313 (4)	0.0558 (3)	0.0156 (8)	
H23A	0.138 (7)	0.511 (6)	−0.005 (6)	0.023*	
H23B	0.032 (7)	0.540 (6)	0.087 (6)	0.023*	
O24	0.1349 (4)	0.3728 (4)	0.3060 (4)	0.0203 (9)	
H24A	0.155 (7)	0.334 (6)	0.368 (6)	0.030*	
H24B	0.143 (8)	0.423 (7)	0.300 (7)	0.030*	
O25	0.3957 (4)	0.1204 (3)	0.1540 (4)	0.0187 (8)	
H25	0.390 (7)	0.070 (6)	0.137 (6)	0.022*	
O26	0.1374 (4)	0.2320 (4)	0.0943 (4)	0.0260 (9)	
H26	0.161 (7)	0.230 (6)	0.018 (6)	0.031*	
O27	0.0421 (4)	−0.0116 (4)	0.3221 (4)	0.0269 (10)	
H27	0.101 (7)	−0.057 (7)	0.313 (7)	0.032*	
O28	−0.0593 (4)	−0.2184 (4)	0.2434 (4)	0.0249 (9)	
H28A	−0.002 (8)	−0.277 (7)	0.247 (7)	0.037*	
H28B	−0.118 (8)	−0.237 (7)	0.254 (7)	0.037*	
O29	0.7568 (4)	0.0498 (4)	0.2733 (4)	0.0182 (8)	
H29	0.735 (6)	0.119 (6)	0.287 (6)	0.022*	
N1	0.6651 (4)	0.0491 (4)	0.0932 (4)	0.0135 (9)	
H1A	0.620503	0.082305	0.041998	0.020*	
H1B	0.694572	−0.035013	0.100535	0.020*	
H1C	0.734329	0.074419	0.066187	0.020*	
N2	−0.0763 (4)	0.3259 (4)	0.3009 (4)	0.0123 (9)	
H2A	−0.109556	0.387738	0.249053	0.018*	
H2B	−0.004148	0.332753	0.301037	0.018*	
H2C	−0.137617	0.331930	0.373678	0.018*	
C1	0.4520 (5)	0.0653 (5)	0.2438 (5)	0.0165 (11)	
H1D	0.472355	−0.025201	0.257484	0.020*	
H1E	0.388654	0.099437	0.317357	0.020*	
C2	0.5768 (5)	0.0910 (4)	0.2092 (4)	0.0107 (10)	
H2	0.554428	0.182003	0.203335	0.013*	
C3	0.6425 (5)	0.0233 (5)	0.2991 (5)	0.0162 (11)	
H3A	0.580025	0.047163	0.376761	0.019*	
H3B	0.667484	−0.066948	0.302642	0.019*	
C4	0.0445 (5)	0.1009 (4)	0.3328 (5)	0.0147 (11)	
H4A	0.135127	0.100118	0.299111	0.018*	
H4B	0.008737	0.113215	0.415968	0.018*	
C5	−0.0397 (5)	0.2030 (4)	0.2660 (4)	0.0127 (10)	
H5	−0.122474	0.187396	0.290152	0.015*	
C6	0.0209 (5)	0.2060 (5)	0.1362 (5)	0.0187 (11)	
H6A	0.041210	0.124953	0.115096	0.022*	
H6B	−0.041824	0.270265	0.098737	0.022*	
O30B	0.9054 (14)	0.105 (2)	−0.080 (3)	0.048 (6)	0.48 (5)
O30A	0.8950 (11)	0.1308 (12)	−0.026 (2)	0.035 (5)	0.52 (5)

### Atomic displacement parameters (Å^2^)

**Table:**

	*U*^11^	*U*^22^	*U*^33^	*U*^12^	*U*^13^	*U*^23^
W1	0.00509 (9)	0.00748 (9)	0.00526 (10)	−0.00281 (7)	−0.00147 (7)	0.00021 (7)
W2	0.00343 (9)	0.00618 (9)	0.00481 (10)	−0.00225 (7)	−0.00065 (7)	−0.00056 (7)
W3	0.00345 (9)	0.00601 (9)	0.00437 (10)	−0.00258 (7)	−0.00026 (7)	−0.00063 (7)
W4	0.00401 (9)	0.00709 (9)	0.00488 (10)	−0.00339 (7)	0.00000 (7)	−0.00063 (7)
W5	0.00430 (9)	0.00760 (9)	0.00575 (10)	−0.00427 (7)	−0.00078 (7)	−0.00051 (7)
W6	0.00366 (9)	0.00720 (9)	0.00671 (10)	−0.00237 (7)	−0.00130 (7)	−0.00083 (7)
Na1	0.0206 (11)	0.0287 (12)	0.0124 (10)	−0.0171 (10)	−0.0008 (9)	−0.0044 (9)
Na2	0.0148 (10)	0.0173 (10)	0.0248 (12)	−0.0047 (9)	−0.0068 (9)	−0.0020 (9)
Na3	0.0135 (10)	0.0150 (10)	0.0215 (11)	−0.0074 (8)	−0.0073 (9)	0.0029 (8)
O1	0.0080 (16)	0.0132 (17)	0.0082 (17)	−0.0048 (14)	0.0000 (13)	−0.0018 (13)
O2	0.0094 (16)	0.0104 (16)	0.0090 (17)	−0.0048 (14)	−0.0031 (14)	−0.0011 (13)
O3	0.0143 (18)	0.0096 (16)	0.0126 (18)	−0.0055 (14)	−0.0058 (15)	0.0012 (13)
O4	0.0070 (16)	0.0101 (16)	0.0045 (16)	−0.0027 (13)	−0.0010 (13)	−0.0010 (13)
O5	0.0052 (15)	0.0071 (15)	0.0071 (16)	−0.0013 (13)	−0.0026 (13)	−0.0001 (12)
O6	0.0111 (17)	0.0153 (17)	0.0043 (16)	−0.0079 (14)	−0.0004 (13)	−0.0017 (13)
O7	0.0098 (17)	0.0085 (16)	0.0120 (18)	−0.0016 (14)	−0.0022 (14)	−0.0008 (13)
O8	0.0032 (15)	0.0079 (15)	0.0069 (16)	−0.0031 (13)	−0.0001 (13)	−0.0012 (12)
O9	0.0036 (15)	0.0071 (15)	0.0088 (16)	−0.0021 (13)	−0.0003 (13)	−0.0013 (12)
O10	0.0017 (15)	0.0078 (15)	0.0059 (16)	−0.0012 (12)	0.0006 (12)	−0.0019 (12)
O11	0.0103 (17)	0.0112 (16)	0.0078 (17)	−0.0075 (14)	−0.0010 (14)	0.0006 (13)
O12	0.0072 (16)	0.0120 (16)	0.0103 (18)	−0.0029 (14)	−0.0008 (14)	−0.0017 (13)
O13	0.0056 (16)	0.0111 (16)	0.0066 (16)	−0.0041 (13)	−0.0014 (13)	−0.0009 (13)
O14	0.0071 (16)	0.0094 (16)	0.0085 (17)	−0.0042 (13)	−0.0022 (13)	−0.0011 (13)
O15	0.0125 (17)	0.0122 (17)	0.0109 (17)	−0.0074 (14)	−0.0018 (14)	−0.0011 (13)
O16	0.0040 (15)	0.0124 (16)	0.0095 (17)	−0.0036 (13)	0.0010 (13)	−0.0029 (13)
O17	0.0076 (16)	0.0137 (16)	0.0076 (17)	−0.0069 (14)	−0.0009 (13)	−0.0027 (13)
O18	0.0126 (17)	0.0139 (17)	0.0102 (17)	−0.0105 (15)	−0.0005 (14)	−0.0012 (14)
O19	0.0063 (16)	0.0088 (16)	0.0090 (17)	−0.0021 (13)	−0.0022 (13)	0.0011 (13)
O20	0.0071 (16)	0.0113 (16)	0.0126 (18)	−0.0037 (14)	−0.0027 (14)	−0.0018 (13)
O21	0.0102 (17)	0.0154 (17)	0.0099 (17)	−0.0070 (14)	−0.0016 (14)	−0.0026 (14)
O22	0.0098 (19)	0.033 (2)	0.015 (2)	−0.0058 (18)	0.0002 (16)	−0.0137 (18)
O23	0.0132 (19)	0.026 (2)	0.0117 (19)	−0.0114 (17)	−0.0014 (16)	−0.0046 (16)
O24	0.027 (2)	0.024 (2)	0.019 (2)	−0.0165 (19)	−0.0117 (18)	0.0022 (18)
O25	0.019 (2)	0.0128 (18)	0.028 (2)	−0.0040 (16)	−0.0139 (17)	−0.0027 (16)
O26	0.031 (2)	0.039 (2)	0.013 (2)	−0.024 (2)	0.0030 (18)	−0.0063 (18)
O27	0.013 (2)	0.012 (2)	0.052 (3)	−0.0004 (16)	−0.010 (2)	−0.0046 (19)
O28	0.013 (2)	0.028 (2)	0.033 (2)	−0.0048 (18)	−0.0076 (19)	−0.0059 (19)
O29	0.0168 (19)	0.0132 (18)	0.032 (2)	−0.0031 (16)	−0.0137 (17)	−0.0076 (16)
N1	0.018 (2)	0.010 (2)	0.010 (2)	−0.0072 (18)	−0.0023 (18)	0.0010 (16)
N2	0.008 (2)	0.015 (2)	0.011 (2)	−0.0023 (17)	−0.0015 (17)	−0.0014 (17)
C1	0.014 (3)	0.022 (3)	0.015 (3)	−0.008 (2)	−0.005 (2)	−0.002 (2)
C2	0.010 (2)	0.007 (2)	0.013 (3)	−0.0018 (19)	−0.003 (2)	−0.0036 (19)
C3	0.019 (3)	0.013 (2)	0.017 (3)	−0.005 (2)	−0.008 (2)	−0.001 (2)
C4	0.014 (3)	0.013 (2)	0.016 (3)	−0.003 (2)	−0.005 (2)	−0.003 (2)
C5	0.005 (2)	0.016 (2)	0.018 (3)	−0.006 (2)	−0.002 (2)	−0.003 (2)
C6	0.016 (3)	0.021 (3)	0.022 (3)	−0.004 (2)	−0.012 (2)	−0.003 (2)
O30B	0.048 (7)	0.092 (10)	0.028 (11)	−0.052 (7)	−0.011 (7)	0.000 (9)
O30A	0.046 (6)	0.048 (7)	0.027 (10)	−0.030 (5)	−0.017 (5)	0.001 (5)

### Geometric parameters (Å, º)

**Table:**

W1—O1	1.743 (3)	Na2—O25	2.321 (4)
W1—O2	1.906 (3)	Na2—O26	2.328 (5)
W1—O3	1.742 (3)	Na3—O7^iv^	2.684 (4)
W1—O4	1.914 (3)	Na3—O18^v^	2.411 (4)
W1—O5	2.274 (3)	Na3—O20^vi^	2.458 (4)
W1—O13^i^	2.270 (3)	Na3—O27	2.375 (5)
W2—O5	1.905 (3)	Na3—O28	2.522 (5)
W2—O6	1.818 (3)	Na3—O29^vii^	2.434 (4)
W2—O7	1.723 (3)	O22—H22A	0.68 (7)
W2—O8	2.259 (3)	O22—H22B	0.90 (7)
W2—O9	2.047 (3)	O23—H23A	0.78 (7)
W2—O10	1.956 (3)	O23—H23B	0.91 (7)
W3—O8	2.255 (3)	O24—H24A	0.86 (7)
W3—O10	1.953 (3)	O24—H24B	0.63 (7)
W3—O11	1.807 (3)	O25—H25	0.73 (7)
W3—O12	1.723 (3)	O25—C1	1.422 (7)
W3—O13	1.896 (3)	O26—H26	0.89 (7)
W3—O14	2.052 (3)	O26—C6	1.420 (7)
W4—O4	1.957 (3)	O27—H27	0.67 (7)
W4—O5	2.227 (3)	O27—C4	1.418 (6)
W4—O11^i^	2.140 (3)	O28—H28A	0.75 (8)
W4—O15	1.761 (3)	O28—H28B	0.79 (8)
W4—O16	1.757 (3)	O29—H29	0.81 (7)
W4—O17	1.858 (3)	O29—C3	1.432 (6)
W5—O8	2.251 (3)	N1—H1A	0.9100
W5—O9	1.845 (3)	N1—H1B	0.9100
W5—O14	1.856 (3)	N1—H1C	0.9100
W5—O17	1.980 (3)	N1—C2	1.489 (6)
W5—O18	1.734 (3)	N2—H2A	0.9100
W5—O19	1.965 (3)	N2—H2B	0.9100
W6—O2^i^	1.966 (3)	N2—H2C	0.9100
W6—O6^i^	2.189 (3)	N2—C5	1.498 (6)
W6—O13	2.228 (3)	C1—H1D	0.9900
W6—O19	1.864 (3)	C1—H1E	0.9900
W6—O20	1.731 (3)	C1—C2	1.525 (7)
W6—O21	1.765 (3)	C2—H2	1.0000
Na1—Na2	3.513 (3)	C2—C3	1.502 (7)
Na1—O1	2.365 (4)	C3—H3A	0.9900
Na1—O12^ii^	2.360 (4)	C3—H3B	0.9900
Na1—O21^i^	2.440 (4)	C4—H4A	0.9900
Na1—O22	2.339 (5)	C4—H4B	0.9900
Na1—O23	2.365 (4)	C4—C5	1.517 (7)
Na1—O30B^iii^	2.96 (3)	C5—H5	1.0000
Na1—O30A^iii^	2.668 (12)	C5—C6	1.496 (7)
Na2—O4^iii^	2.606 (4)	C6—H6A	0.9900
Na2—O22	2.299 (4)	C6—H6B	0.9900
Na2—O23	2.471 (4)	O30B—O30A	0.772 (14)
Na2—O24	2.409 (5)		
			
O1—W1—O2	99.34 (14)	O24—Na2—Na1	82.89 (12)
O1—W1—O4	97.41 (14)	O24—Na2—O4^iii^	163.49 (15)
O1—W1—O5	165.62 (13)	O24—Na2—H22A	99.6 (17)
O1—W1—O13^i^	90.34 (13)	O24—Na2—O23	83.19 (15)
O2—W1—O4	152.13 (14)	O24—Na2—H23A	99.9 (16)
O2—W1—O5	85.31 (13)	O24—Na2—H24B	14.0 (16)
O2—W1—O13^i^	72.81 (12)	O25—Na2—Na1	141.81 (13)
O3—W1—O1	101.50 (15)	O25—Na2—O4^iii^	95.66 (14)
O3—W1—O2	97.16 (15)	O25—Na2—H22A	91.2 (16)
O3—W1—O4	101.13 (15)	O25—Na2—O23	175.41 (17)
O3—W1—O5	91.33 (14)	O25—Na2—H23A	158.6 (16)
O3—W1—O13^i^	165.73 (14)	O25—Na2—O24	99.07 (17)
O4—W1—O5	73.49 (12)	O25—Na2—H24B	107.6 (17)
O4—W1—O13^i^	85.00 (12)	O25—Na2—O26	84.84 (16)
O13^i^—W1—O5	77.96 (11)	O26—Na2—Na1	133.01 (14)
O5—W2—O8	85.93 (12)	O26—Na2—O4^iii^	91.27 (14)
O5—W2—O9	84.55 (13)	O26—Na2—H22A	162.9 (17)
O5—W2—O10	155.42 (13)	O26—Na2—O23	90.92 (16)
O6—W2—O5	95.34 (14)	O26—Na2—H23A	82.9 (15)
O6—W2—O8	88.22 (13)	O26—Na2—O24	97.41 (16)
O6—W2—O9	160.42 (14)	O26—Na2—H24B	108.9 (17)
O6—W2—O10	91.33 (13)	O18^v^—Na3—O7^iv^	99.45 (13)
O7—W2—O5	104.66 (15)	O18^v^—Na3—O20^vi^	82.68 (13)
O7—W2—O6	103.57 (16)	O18^v^—Na3—O28	79.23 (14)
O7—W2—O8	163.09 (14)	O18^v^—Na3—O29^vii^	104.03 (14)
O7—W2—O9	95.34 (14)	O20^vi^—Na3—O7^iv^	118.30 (13)
O7—W2—O10	96.66 (14)	O20^vi^—Na3—O28	82.88 (14)
O9—W2—O8	72.23 (11)	O27—Na3—O7^iv^	88.69 (14)
O10—W2—O8	70.63 (12)	O27—Na3—O18^v^	162.22 (16)
O10—W2—O9	81.39 (13)	O27—Na3—O20^vi^	79.54 (14)
O10—W3—O8	70.79 (12)	O27—Na3—O28	98.85 (17)
O10—W3—O14	82.41 (13)	O27—Na3—O29^vii^	92.51 (16)
O11—W3—O8	87.05 (13)	O28—Na3—O7^iv^	158.61 (15)
O11—W3—O10	92.51 (14)	O29^vii^—Na3—O7^iv^	83.56 (13)
O11—W3—O13	94.39 (14)	O29^vii^—Na3—O20^vi^	156.19 (16)
O11—W3—O14	160.06 (14)	O29^vii^—Na3—O28	76.17 (15)
O12—W3—O8	164.22 (13)	W1—O1—Na1	135.79 (18)
O12—W3—O10	97.08 (14)	W1—O2—W6^i^	117.99 (16)
O12—W3—O11	103.95 (15)	W1—O4—W4	117.48 (16)
O12—W3—O13	103.07 (15)	W1—O4—Na2^iii^	116.67 (15)
O12—W3—O14	95.83 (14)	W4—O4—Na2^iii^	116.38 (15)
O13—W3—O8	87.06 (13)	W2—O5—W1	125.33 (15)
O13—W3—O10	156.41 (13)	W2—O5—W4	137.59 (16)
O13—W3—O14	83.45 (13)	W4—O5—W1	94.63 (12)
O14—W3—O8	73.06 (12)	W2—O6—W6^i^	139.38 (17)
O4—W4—O5	73.85 (12)	W2—O7—Na3^iv^	137.29 (18)
O4—W4—O11^i^	81.97 (13)	W3—O8—W2	97.22 (12)
O11^i^—W4—O5	77.76 (12)	W5—O8—W2	94.75 (11)
O15—W4—O4	92.61 (14)	W5—O8—W3	94.52 (12)
O15—W4—O5	96.44 (14)	W5—O9—W2	116.91 (15)
O15—W4—O11^i^	172.94 (14)	W3—O10—W2	120.08 (15)
O15—W4—O17	96.69 (15)	W3—O11—W4^i^	136.32 (18)
O16—W4—O4	96.54 (14)	W3—O12—Na1^viii^	170.67 (19)
O16—W4—O5	160.85 (13)	W3—O13—W1^i^	126.28 (15)
O16—W4—O11^i^	84.60 (13)	W3—O13—W6	137.29 (16)
O16—W4—O15	100.58 (15)	W6—O13—W1^i^	95.12 (12)
O16—W4—O17	99.46 (15)	W5—O14—W3	115.64 (16)
O17—W4—O4	159.66 (14)	W4—O17—W5	148.36 (18)
O17—W4—O5	87.12 (13)	W5—O18—Na3^ix^	150.89 (19)
O17—W4—O11^i^	87.11 (13)	W6—O19—W5	146.16 (18)
O9—W5—O8	76.09 (12)	W6—O20—Na3^vi^	132.86 (18)
O9—W5—O14	93.33 (14)	W6—O21—Na1^i^	135.54 (18)
O9—W5—O17	86.47 (14)	Na1—O22—H22A	122 (6)
O9—W5—O19	154.74 (14)	Na1—O22—H22B	114 (4)
O14—W5—O8	76.78 (12)	Na2—O22—Na1	98.47 (16)
O14—W5—O17	156.72 (13)	Na2—O22—H22A	104 (6)
O14—W5—O19	87.83 (14)	Na2—O22—H22B	113 (4)
O17—W5—O8	80.60 (12)	H22A—O22—H22B	104 (7)
O18—W5—O8	178.51 (14)	Na1—O23—Na2	93.15 (15)
O18—W5—O9	102.80 (15)	Na1—O23—H23A	113 (5)
O18—W5—O14	102.35 (15)	Na1—O23—H23B	138 (4)
O18—W5—O17	100.38 (14)	Na2—O23—H23A	88 (5)
O18—W5—O19	101.57 (15)	Na2—O23—H23B	113 (4)
O19—W5—O8	79.64 (12)	H23A—O23—H23B	101 (6)
O19—W5—O17	82.74 (13)	Na2—O24—H24A	126 (5)
O2^i^—W6—O6^i^	77.66 (13)	Na2—O24—H24B	98 (7)
O2^i^—W6—O13	72.73 (12)	H24A—O24—H24B	101 (8)
O6^i^—W6—O13	76.98 (12)	Na2—O25—H25	118 (5)
O19—W6—O2^i^	156.59 (14)	C1—O25—Na2	130.9 (3)
O19—W6—O6^i^	84.75 (13)	C1—O25—H25	107 (5)
O19—W6—O13	88.45 (13)	Na2—O26—H26	106 (4)
O20—W6—O2^i^	94.54 (14)	C6—O26—Na2	150.9 (3)
O20—W6—O6^i^	90.10 (14)	C6—O26—H26	100 (4)
O20—W6—O13	163.39 (13)	Na3—O27—H27	110 (6)
O20—W6—O19	100.87 (15)	C4—O27—Na3	131.5 (3)
O20—W6—O21	101.60 (16)	C4—O27—H27	112 (6)
O21—W6—O2^i^	95.80 (14)	Na3—O28—H28A	110 (6)
O21—W6—O6^i^	167.09 (14)	Na3—O28—H28B	107 (6)
O21—W6—O13	90.50 (14)	H28A—O28—H28B	108 (8)
O21—W6—O19	98.20 (15)	Na3^x^—O29—H29	108 (5)
O1—Na1—Na2	130.03 (12)	C3—O29—Na3^x^	107.8 (3)
O1—Na1—O21^i^	87.94 (13)	C3—O29—H29	109 (5)
O1—Na1—O30B^iii^	75.4 (4)	H1A—N1—H1B	109.5
O1—Na1—O30A^iii^	82.9 (4)	H1A—N1—H1C	109.5
O12^ii^—Na1—Na2	90.80 (11)	H1B—N1—H1C	109.5
O12^ii^—Na1—O1	91.95 (13)	C2—N1—H1A	109.5
O12^ii^—Na1—O21^i^	169.87 (16)	C2—N1—H1B	109.5
O12^ii^—Na1—O23	87.84 (14)	C2—N1—H1C	109.5
O12^ii^—Na1—O30B^iii^	106.9 (5)	H2A—N2—H2B	109.5
O12^ii^—Na1—O30A^iii^	94.4 (6)	H2A—N2—H2C	109.5
O21^i^—Na1—Na2	81.56 (10)	H2B—N2—H2C	109.5
O21^i^—Na1—O30B^iii^	82.9 (6)	C5—N2—H2A	109.5
O21^i^—Na1—O30A^iii^	95.6 (6)	C5—N2—H2B	109.5
O22—Na1—Na2	40.33 (11)	C5—N2—H2C	109.5
O22—Na1—O1	89.70 (15)	O25—C1—H1D	109.6
O22—Na1—O12^ii^	92.67 (15)	O25—C1—H1E	109.6
O22—Na1—O21^i^	77.19 (14)	O25—C1—C2	110.2 (4)
O22—Na1—O23	84.94 (15)	H1D—C1—H1E	108.1
O22—Na1—O30B^iii^	155.5 (6)	C2—C1—H1D	109.6
O22—Na1—O30A^iii^	169.9 (6)	C2—C1—H1E	109.6
O23—Na1—Na2	44.61 (11)	N1—C2—C1	108.4 (4)
O23—Na1—O1	174.62 (17)	N1—C2—H2	109.3
O23—Na1—O21^i^	91.32 (14)	N1—C2—C3	110.2 (4)
O23—Na1—O30B^iii^	109.8 (4)	C1—C2—H2	109.3
O23—Na1—O30A^iii^	102.5 (4)	C3—C2—C1	110.2 (4)
O30B^iii^—Na1—Na2	149.3 (2)	C3—C2—H2	109.3
O30A^iii^—Na1—Na2	146.5 (3)	O29—C3—C2	112.1 (4)
O30A^iii^—Na1—O30B^iii^	14.6 (2)	O29—C3—H3A	109.2
Na1—Na2—H22A	51.3 (16)	O29—C3—H3B	109.2
Na1—Na2—H23A	51.3 (15)	C2—C3—H3A	109.2
Na1—Na2—H24B	69.2 (17)	C2—C3—H3B	109.2
O4^iii^—Na2—Na1	80.92 (9)	H3A—C3—H3B	107.9
O4^iii^—Na2—H22A	72.6 (17)	O27—C4—H4A	110.5
O4^iii^—Na2—H23A	67.2 (16)	O27—C4—H4B	110.5
O4^iii^—Na2—H24B	150.2 (17)	O27—C4—C5	106.2 (4)
O22—Na2—Na1	41.20 (11)	H4A—C4—H4B	108.7
O22—Na2—O4^iii^	83.35 (14)	C5—C4—H4A	110.5
O22—Na2—H22A	15.0 (16)	C5—C4—H4B	110.5
O22—Na2—O23	83.43 (15)	N2—C5—C4	110.1 (4)
O22—Na2—H23A	90.4 (15)	N2—C5—H5	107.2
O22—Na2—O24	86.63 (15)	C4—C5—H5	107.2
O22—Na2—H24B	74.3 (17)	C6—C5—N2	111.0 (4)
O22—Na2—O25	100.64 (16)	C6—C5—C4	113.9 (4)
O22—Na2—O26	172.64 (19)	C6—C5—H5	107.2
H22A—Na2—H23A	95 (2)	O26—C6—C5	110.7 (4)
H22A—Na2—H24B	88 (2)	O26—C6—H6A	109.5
O23—Na2—Na1	42.24 (10)	O26—C6—H6B	109.5
O23—Na2—O4^iii^	82.65 (13)	C5—C6—H6A	109.5
O23—Na2—H22A	92.4 (16)	C5—C6—H6B	109.5
O23—Na2—H23A	17.7 (15)	H6A—C6—H6B	108.1
O23—Na2—H24B	75.5 (17)	O30A—O30B—Na1^iii^	60.3 (18)
H23A—Na2—H24B	93 (2)	O30B—O30A—Na1^iii^	105 (2)
			
Na2—O25—C1—C2	−81.3 (5)	O11^i^—W4—O17—W5	−70.5 (3)
Na2—O26—C6—C5	−28.2 (9)	O12—W3—O11—W4^i^	−55.6 (3)
Na3—O27—C4—C5	−70.6 (6)	O12—W3—O13—W1^i^	60.5 (2)
Na3^x^—O29—C3—C2	−169.3 (3)	O12—W3—O13—W6	−136.2 (2)
O2—W1—O1—Na1	−25.9 (3)	O13^i^—W1—O1—Na1	46.8 (3)
O2^i^—W6—O19—W5	47.2 (6)	O13—W3—O11—W4^i^	49.1 (3)
O2^i^—W6—O20—Na3^vi^	−50.9 (2)	O13—W6—O19—W5	11.3 (3)
O2^i^—W6—O21—Na1^i^	−30.6 (3)	O13—W6—O20—Na3^vi^	−90.0 (5)
O3—W1—O1—Na1	−125.2 (3)	O13—W6—O21—Na1^i^	42.1 (3)
O4—W1—O1—Na1	131.8 (2)	O14—W3—O11—W4^i^	132.0 (3)
O4—W4—O17—W5	−13.1 (6)	O14—W3—O13—W1^i^	155.0 (2)
O5—W1—O1—Na1	82.1 (6)	O14—W3—O13—W6	−41.7 (2)
O5—W2—O6—W6^i^	−42.3 (3)	O14—W5—O9—W2	−76.39 (18)
O5—W2—O7—Na3^iv^	−150.8 (2)	O14—W5—O18—Na3^ix^	−153.2 (4)
O5—W4—O17—W5	7.4 (3)	O15—W4—O17—W5	103.5 (3)
O6—W2—O7—Na3^iv^	109.9 (2)	O16—W4—O17—W5	−154.6 (3)
O6^i^—W6—O19—W5	88.4 (3)	O17—W5—O9—W2	80.29 (17)
O6^i^—W6—O20—Na3^vi^	−128.5 (2)	O17—W5—O14—W3	−13.9 (4)
O6^i^—W6—O21—Na1^i^	28.1 (8)	O17—W5—O18—Na3^ix^	31.8 (4)
O7—W2—O6—W6^i^	64.3 (3)	O18—W5—O9—W2	−179.85 (17)
O8—W2—O6—W6^i^	−128.0 (3)	O18—W5—O14—W3	178.84 (17)
O8—W2—O7—Na3^iv^	−23.3 (6)	O19—W5—O9—W2	15.6 (4)
O8—W3—O11—W4^i^	135.9 (2)	O19—W5—O14—W3	−79.79 (17)
O8—W3—O13—W1^i^	−131.77 (19)	O19—W5—O18—Na3^ix^	116.5 (4)
O8—W3—O13—W6	31.6 (2)	O19—W6—O20—Na3^vi^	146.9 (2)
O8—W5—O9—W2	−0.89 (15)	O19—W6—O21—Na1^i^	130.6 (2)
O8—W5—O14—W3	0.08 (15)	O20—W6—O19—W5	177.5 (3)
O9—W2—O6—W6^i^	−131.0 (3)	O20—W6—O21—Na1^i^	−126.5 (3)
O9—W2—O7—Na3^iv^	−65.0 (3)	O21—W6—O19—W5	−79.0 (3)
O9—W5—O14—W3	74.95 (18)	O21—W6—O20—Na3^vi^	46.0 (3)
O9—W5—O18—Na3^ix^	−56.9 (4)	O25—C1—C2—N1	−54.2 (5)
O10—W2—O6—W6^i^	161.4 (3)	O25—C1—C2—C3	−174.9 (4)
O10—W2—O7—Na3^iv^	16.9 (3)	O27—C4—C5—N2	163.1 (4)
O10—W3—O11—W4^i^	−153.5 (2)	O27—C4—C5—C6	−71.6 (5)
O10—W3—O13—W1^i^	−151.6 (2)	N1—C2—C3—O29	63.4 (5)
O10—W3—O13—W6	11.8 (5)	N2—C5—C6—O26	61.3 (5)
O11—W3—O13—W1^i^	−45.0 (2)	C1—C2—C3—O29	−177.0 (4)
O11—W3—O13—W6	118.4 (2)	C4—C5—C6—O26	−63.6 (6)

Symmetry codes: (i) −*x*+1, −*y*+1, −*z*+1; (ii) *x*, *y*, *z*−1; (iii) −*x*+1, −*y*+1, −*z*; (iv) −*x*, −*y*+1, −*z*+1; (v) *x*−1, *y*−1, *z*; (vi) −*x*+1, −*y*, −*z*+1; (vii) *x*−1, *y*, *z*; (viii) *x*, *y*, *z*+1; (ix) *x*+1, *y*+1, *z*; (x) *x*+1, *y*, *z*.

### Hydrogen-bond geometry (Å, º)

**Table:**

*D*—H···*A*	*D*—H	H···*A*	*D*···*A*	*D*—H···*A*
O22—H22*A*···O1^iii^	0.67 (7)	2.11 (7)	2.757 (5)	163 (8)
O23—H23*A*···O16^iii^	0.78 (7)	2.04 (7)	2.802 (5)	165 (7)
O23—H23*B*···O16^vii^	0.91 (7)	1.91 (7)	2.811 (5)	175 (6)
O25—H25···O3^xi^	0.73 (6)	2.05 (7)	2.761 (5)	164 (7)
O26—H26···O15^iii^	0.88 (7)	1.88 (7)	2.730 (5)	160 (6)
O27—H27···O2^xi^	0.67 (7)	2.04 (7)	2.696 (5)	165 (8)
O28—H28*A*···O21^vi^	0.75 (8)	2.01 (8)	2.735 (6)	160 (8)
O29—H29···O10^i^	0.81 (6)	1.91 (7)	2.692 (5)	163 (6)
N1—H1*B*···O15^xi^	0.91	1.82	2.723 (5)	170
N2—H2*B*···O24	0.91	1.96	2.867 (6)	172

Symmetry codes: (i) −*x*+1, −*y*+1, −*z*+1; (iii) −*x*+1, −*y*+1, −*z*; (vi) −*x*+1, −*y*, −*z*+1; (vii) *x*−1, *y*, *z*; (xi) *x*, *y*−1, *z*.
